# A Robust and Low Computational Cost Pitch Estimation Method

**DOI:** 10.3390/s22166026

**Published:** 2022-08-12

**Authors:** Desheng Wang, Yangjie Wei, Yi Wang, Jing Wang

**Affiliations:** 1 Key Laboratory of Intelligent Computing in Medical Image, Ministry of Education, School of Computer Science and Engineering, Northeastern University, Shenyang 110169, China; 2 School of Information Science and Engineering, Shenyang University of Technology, Shenyang 110870, China

**Keywords:** pitch estimation, harmonic structure, harmonic summation (HS), smooth prior

## Abstract

Pitch estimation is widely used in speech and audio signal processing. However, the current methods of modeling harmonic structure used for pitch estimation cannot always match the harmonic distribution of actual signals. Due to the structure of vocal tract, the acoustic nature of musical equipment, and the spectrum leakage issue, speech and audio signals’ harmonic frequencies often slightly deviate from the integer multiple of the pitch. This paper starts with the summation of residual harmonics (SRH) method and makes two main modifications. First, the spectral peak position constraint of strict integer multiple is modified to allow slight deviation, which benefits capturing harmonics. Second, a main pitch segment extension scheme with low computational cost feature is proposed to utilize the smooth prior of pitch more efficiently. Besides, the pitch segment extension scheme is also integrated into the SRH method’s voiced/unvoiced decision to reduce short-term errors. Accuracy comparison experiments with ten pitch estimation methods show that the proposed method has better overall accuracy and robustness. Time cost experiments show that the time cost of the proposed method reduces to around 1/8 of the state-of-the-art fast NLS method on the experimental computer.

## 1. Introduction

Pitch is a subjective psychoacoustic phenomenon synthesized by the ear auditory cortex system for the brain [1]. As a basic feature, pitch is widely used in the areas of speech interaction [2,3,4,5,6], music signal processing [7,8,9,10,11], and medical diagnosis [12,13]. Research on pitch estimation has been going on for decades, and estimating pitch from clean speech has been considered a solved problem because many methods achieve high estimation accuracy under high signal-to-noise ratio (SNR) conditions. However, the robustness of pitch estimation under noise and reverberation conditions still needs to be improved. Drugman and Alwan of the University of Mons, Belgium, authors of the well-known summation of residual harmonics (SRH) pitch estimation method, emphasize that performance under noisy conditions is the focus of research in pitch estimation over the next decade [14,15].

**Figure 1:**
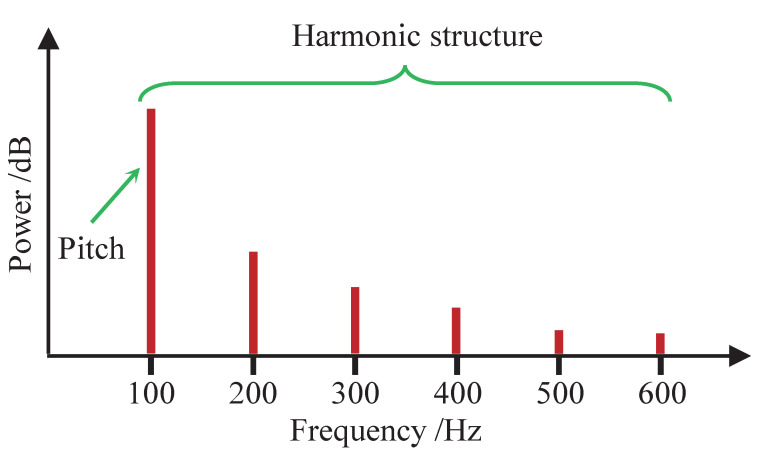
Harmonic structure of audio signal.

The robustness of pitch estimation is affected by the model accuracy of the method, and the modeling of almost all pitch estimation methods directly or indirectly depends on the harmonic structure since the harmonic structure is an essential feature of audio signals. Figure 1 shows the harmonic structure of an audio signal. The spectral peak with a frequency of 100 Hz is the pitch, and the higher spectral peaks located near integer multiples of 100 Hz constitute the harmonic structure of the pitch. A fundamental assumption of modeling harmonic structures used in the pitch estimation is that the harmonic components are strictly distributed at integer multiples of the pitch [14,16,17,18]. Expressed in a formula, this modeling method on harmonic structures is generally realized by the product of an integer and the pitch, that is:

(1) $$f_{l}=lf_{0}\;\left(l=2,\ldots L\right)$$

The harmonic summation (HS)-based methods such as SRH and subharmonic-to-harmonic ratio (SHR), are a typical category of methods using (1) to select the harmonic components directly. Besides, (1) also exists in the commonly used harmonic models, as follows, wherein parameters *l* in (2) and (3) correspond to the strict integer multiple relationship between the pitch and its harmonics.

(1) Harmonic model or harmonic plus noise model [12,19], which can be denoted by:

(2) $$\left\{\begin{array}{c} x\left(n\right)=\sum_{l=1}^{L}A_{l}\cos\left(l\omega_{0}n+\Phi_{l}\right)+e\left(n\right)\\ x\left(n\right)=\sum_{l=1}^{L}[a_{l}\cos\left(l\omega_{0}n\right)-b_{l}\sin\left(l\omega_{0}n\right)]+e\left(n\right) \end{array}\right.$$

where x(n) is the discrete time signal, including noise sequence e(n); $A_{l}$, $a_{l}$, $b_{l}$, and $\Phi_{l}$ are the linear weights and the initial phase of the $l^{th}$ harmonic, respectively; $\omega_{0}=2\pi f_{0}/f_{s}$ is the normalized angular frequency in radians; $f_{s}$ is the sampling rate. The second part of (2) is one equivalent example of the first part of (2), and it is derived using the trigonometric product-to-sum formula.

(2) Tied Gaussian mixture model (tied-GMM) [20], within which each harmonic is assumed to be a frequency probability distribution approximated with a Gaussian distribution. In log-frequency scale, integer multiples of harmonics correspond to addition operations. Thus, the means of the tied-GMM are represented by:

(3) u={u,…,u+logl,…,u+logL}

where *u* corresponds to the pitch $f_{0}$, and *l* denotes the index of the harmonic.

**Figure 2:**
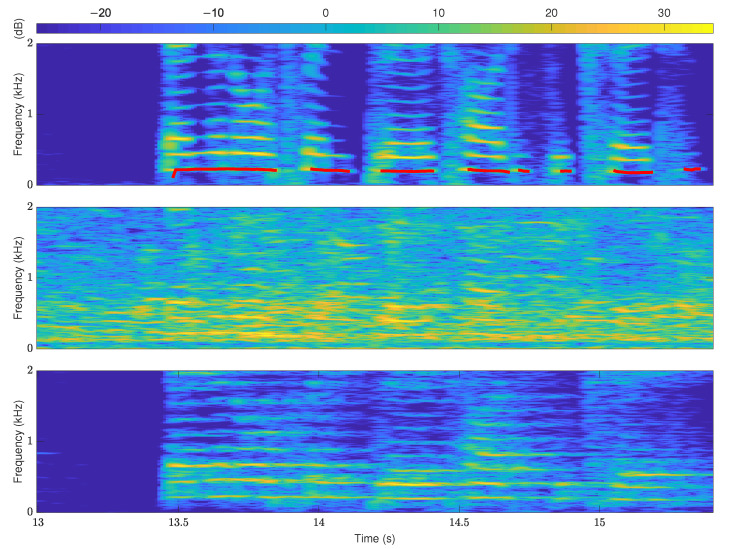
Smooth prior of pitch trajectory. The spectrogram corresponds to a segment of speech signal named “f1nw0000.pes” in the Keele database. The upper one is clean speech, wherein the red lines are pitch trajectories. The middle one is under babble noise of −5 dB SNR. The lower one is under RT60 = 1.0 s, and the other conditions are the same as the subsequent experiment.

However, the assumption that the harmonic structure has a strict integer multiple relationship with the pitch does not always hold in practice. The effects of the structure of the vocal tract, the acoustic nature of musical equipment, and the spectral leakage issue may all cause harmonic components to be shifted relative to integer frequency positions. In addition to the above-mentioned accuracy of modeling the harmonic structure affecting the robustness of the pitch estimation, the use of priors by pitch estimation methods also matters. Noise and reverberation can corrupt and distort the harmonic structure of speech signals, and it is necessary to introduce additional priors into pitch estimation methods to improve robustness. The middle and lower parts in Figure 2 correspond to the spectrograms under noise and reverberation conditions, respectively. Obviously, the harmonic structures represented by the bright yellow parts in Figure 2 are ambiguous. It is challenging to obtain high accuracy for pitch estimation methods that rely entirely on harmonic structure. This is also the main reason that the performance of many pitch estimation methods decreases rapidly when the SNR continues to decrease below negative values. Smooth prior is the basic prior knowledge of pitch and provides a constructive method for pitch estimation. The red line in the upper part of Figure 2 is the connection of the pitches of voiced frames. The smooth prior represents that the pitch trajectory is generally continuous and smooth with the time change. The idea of smooth prior has been integrated into the pitch estimation methods to improve the pitch estimation robustness and accuracy in different ways, such as Bayesian [12], Kalman filtering [13], and particle filter [21]. However, the current methods of using smooth prior are still computationally expensive compared to HS, which has been theoretically proven to be a theoretical approximation of the most accurate non-linear least squares (NLS) method [19]. This not only affects the robustness of HS-based pitch estimation methods under noise and reverberation conditions, but also limits the application of pitch estimation in computing-limited scenarios.

This paper’s contribution is to improve pitch estimation’s robustness more efficiently by improving the accuracy of modeling harmonic structure and realizing the smooth prior at a low computational cost. Two improvements are proposed and integrated into the proposed pitch estimation method based on SRH. First, a loose constraint is introduced to make the modeling of harmonic structure more closely match the actual harmonic distribution. Second, the smooth prior is utilized in a low-complexity way by finding the continuous pitch segment with high confidence, and expanding this pitch segment forward and backward, respectively. Besides, the idea of the continuous pitch segment is also integrated into the SRH’s voiced/unvoiced decision to reduce the short-term errors at both ends and in the middle of the voiced segment. Accuracy comparison experiments under noise and reverberation conditions with ten pitch estimation methods show that the proposed method possesses better overall accuracy and robustness. Time cost comparison shows that the time cost of the proposed method reduces to around one-eighth of the state-of-the-art fast NLS method on the experimental computer.

The paper is organized as follows: In Section 2, related works in the literature are briefly introduced. In Section 3, the overall structure of the proposed pitch estimation method is introduced, and the differences relative to the SRH method are highlighted subsequently. In Section 4, experiments under noise and reverberation conditions that influence the accuracy of pitch estimation methods are carried out and analyzed. Finally, the conclusions are summarized in Section 5.

## 2. Related Work

The existing pitch estimation methods can be classified as time-domain methods, frequency-domain methods, mixed-domain methods, and neural network-based methods. YIN and RAPT are well-known time-domain methods that estimate pitch directly from the signal waveform using autocorrelation [22,23]. As a supplement, the cumulative average normalized difference function and some post-processing techniques are used in YIN to improve the accuracy of the autocorrelation. Similarly, RAPT calculates the pitch based on a short-term speech signal’s normalized cross-correlation function (NCCF). The characteristic of RAPT is using two different sampling rates, one at the original sampling rate, and the other at a significantly reduced sampling rate [23]. However, the general problem of time-domain methods is the robustness under low SNR conditions. Comparative experiments in multiple research show that YIN fails rapidly under negative SNRs, and is more sensitive to colored noise [12,15].

In contrast, frequency-domain methods, such as various harmonic summation (HS)-based methods, generally exhibit better robustness. The HS-based methods have the advantage of being a theoretical approximation to the most accurate NLS method, while having a much lower computational complexity [12,19]. The HS-based methods generally obtain pitch candidates by processing the peaks in the power spectrum and select the pitch according to the HS value of the candidates. The differences between the HS-based methods are mainly in the objective function used for summing the power of the harmonics [16,17], or residual harmonics [14]. The original objective function summed the powers of the harmonics directly in [16]. Then, the SHR method revised the objective function as a ratio of the harmonic power summation to the power summation of the subharmonic [17]. This replacement not only measures the harmonic power, but also excludes non-harmonic noise. Further, the summation of residual harmonics (SRH) method used an auto-regressive linear predictive coding (LPC) filter to achieve the function of pre-whitening and the removal of vocal tract effects [14]. Besides, typical frequency-domain methods also include PEFAC [18] and SWIPE [24]. Both the PEFAC and SWIPE can be seen as HS-based methods in a broad sense. SWIPE is a harmonic comb pitch estimation method with cosine-shaped teeth that smoothly connects harmonic peaks with sub-harmonic valleys, and another feature is that it only uses the first few significant harmonics of the signal [24]. The PEFAC realized a harmonic summation filter in the log-frequency power spectral domain [18], and PEFAC’s objective function is similar to the original HS. However, the long frame length requirement of PEFAC makes it inappropriate for time-critical applications, such as in hearing aids [12].

Mixed-domain methods are theoretically more advantageous than time-domain or frequency-domain methods, but this advantage is still not obvious in practice. YAAPT is a typical mixed-domain method with features of nonlinear processing and dynamic programming [25]. Although the accuracy of YAAPT is better than the time-domain methods such as Yin, its gap with the excellent frequency-domain methods is noticeable under low SNR conditions according to the results in [15]. Besides, mixed-domain pitch extractions are also adopted in ETSI extended distributed speech recognition (DSR) standards ES 202 211 and ES 202 212 for server-side voice reconstruction [26,27,28], and in a high-quality speech manipulation system for time interval and frequency cues extraction [29]. Although the pitch estimation method based on a neural network is indeed based on time-domain and/or frequency-domain methods, the use of neural networks makes this category distinctly different. The recently proposed LACOPE is a deep learning-based pitch estimation algorithm, and it is trained in a joint pitch estimation and speech enhancement framework [3]. The feature of LACOPE provides a trade-off between pitch accuracy and latency by allowing for a configurable latency. This feature is achieved by compensating for the delay caused by feature computation by predicting the pitch through a neural network. CREPE is a deep convolutional neural network-based pitch estimation method based on the time-domain signal [30]. CREPE is claimed to be slightly better than the probabilistic successor of the classic YIN in [3]. However, the CREPE needs to be retrained if the user’s frequency resolution or frame length requirement is not the same as the pre-trained model, which can be a very time-consuming process as pointed out in [12].

## 3. Proposed Pitch Estimation Method

The proposed pitch estimation method belongs to the HS-based method, and specifically, it is a variant of the SRH [14]. However, the core formula of modeling the harmonic structure and the scheme of using the smooth prior of the proposed pitch estimation method differs from the HS-based methods and SRH, and these differences contribute to the performance improvement. This section first introduces the overall structure of the proposed pitch estimation method, and then highlights the differences.

### 3.1. Overall Structure of the Proposed Pitch Estimation Method

**Figure 3:**
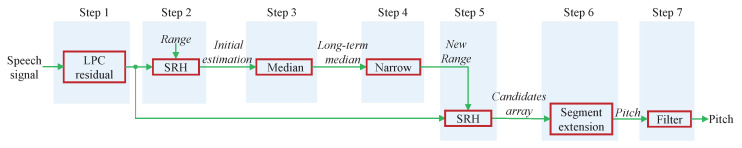
Main steps of the proposed pitch estimation method.

The overall structure of the proposed pitch estimation method is shown in Figure 3. The proposed pitch estimation method mainly includes three portions: (1) whiten the input speech signal through an LPC filter; (2) narrow the target pitch range of the second SRH operation through the initial estimation of the first SRH operation; (3) perform the segment expansion on the candidate pitch array and perform filtering on the pitch trajectory. The three portions correspond to the seven steps in Figure 3.

Step 1: Calculate the speech signal’s residual spectrum by using an auto-regressive LPC filter. This operation is for pre-whitening and removing the effect of the vocal tract, and the LPC filter in the proposed method is the same as that in the original SRH [14]. The data frame length for calculating the spectrum affects the accuracy of pitch estimation. In harsh environments, the signal integrity is severely destroyed. Thus, acquiring more signal periods is necessary for accurate signal identification. The longer the data frame, the more signal power is integrated by the FFT, which benefits the signal extraction from noise. However, a longdata frame length decreases the temporal resolution of pitch changes. Therefore, the data frame length in the proposed method is set to 102.4 ms with 10 ms hop size, which is a trade-off between accuracy and temporal resolution. The FFT length is set to the sampling rate by padding each data frame with zeros, which is inherited from the original SRH method. For the dataset with a 20 kHz sampling rate, 102.4 ms corresponds to 2048 samplings, and the FFT length is achieved by extending the 2048 samples to 20 k by padding zeros. Similar to the original SRH method, resampling is used in the proposed method to avoid overlong FFT and LPC computation under high sampling rate conditions. The input signal is resampled to 16 kHz if the input signal’s sampling rate exceeds 22.05 kHz.

Step 2: for each frame of the residual spectrum, calculate the SRH values of each frequency within the default searching range $Range=[f_{0,\min},f_{0,\max}]$ of pitch based on the residual spectrum. The frequency corresponding to the largest SRH value is selected as the initial estimation of this frame. The default parameter of Range in the proposed method is set to [50, 400], which are commonly used values in practice. Since the formula for calculating the SRH value is the core of the SRH, the formulas in the SRH method and the improved method are separately introduced in the subsequent Section 3.2.

Step 3: apply the median function to the initial estimation of the pitch sequence to get the frequency median $f_{\mathrm{median}}$. This median is used to adjust the subsequent pitch search range.

Step 4: narrow the pitch search range by narrowing the default Range according to the frequency median $f_{\mathrm{median}}$. The constrained parameters 2 and 1/2 are based on the assumption that a normal speaker will not exceed these limits [31].

(4) $$\left\{\begin{array}{c} f_{0,\min}=\max(50,\frac{1}{2}f_{\mathrm{median}})\\ f_{0,\max}=\min(400,2f_{\mathrm{median}}) \end{array}\right.$$

wherein the function max(x,y) and min(x,y) represent selecting the maximal and the minimal value from *x* and *y*, respectively.

Step 5: for each frame of the residual spectrum, recalculate the SRH value of each frequency within the narrowed searching range of pitch based on the residual spectrum. Select the frequencies corresponding to each frame’s largest SRH value as the candidate array’s first row. Besides, select the frequencies that correspond to the second largest SRH value of each frame as the second row of the candidate array. Thus, in addition to selecting the frequency with the largest SRH value, only one candidate frequency is selected for each frame. This is because octave error is a major aspect of SRH estimation error, and when octave error occurs, the frequency with the second largest SRH value is usually the correct pitch.

Step 6: update the pitch sequence from the candidate array by using the segment extension operation. The segment extension operation is added in the proposed pitch estimation relative to the SRH, and the details are introduced in the subsequent Section 3.3.

Step 7: apply a moving median filter to the pitch sequence with a window length of three hops. This post-processing operation helps to improve the accuracy of the pitch estimation method by smoothing the pitch trajectory.

The main steps of the proposed pitch estimation method are described above. The significant differences between the pitch estimation method and the SRH method are the newly added segment expansion operation for reducing pitch jumping, and the revision of the core formula for calculating SRH. Next, we introduce the significant differences in detail.

### 3.2. Modeling Harmonics with Loose Constraint

In the SRH method, assume the specified pitch estimation range is $\left[f_{0,\min},f_{0,\max}\right]$, the SRH value of each frequency within the range is calculated by:

(5) $$SRH\left(f_{0}\right)=P\left(f_{0}\right)+\sum_{l=2}^{L}P\left(lf_{0}\right)-\sum_{l=2}^{L}P\left(\left(l-\frac{1}{2}\right)f_{0}\right)$$

where the integer multiple relationship $lf_{0}$ in (5) equals $f_{l}$ in (1). Parameter *P* denotes the residual spectrum function. The first summation portion in (5) represents the harmonic comb of the SRH method that is based on the integer multiple relationship of harmonic structure. The second summation portion in (5) shows the supplemented harmonic comb with negative teeth at the sub-harmonic that is also based on the integer multiple relationship of harmonic structure.

**Figure 4:**
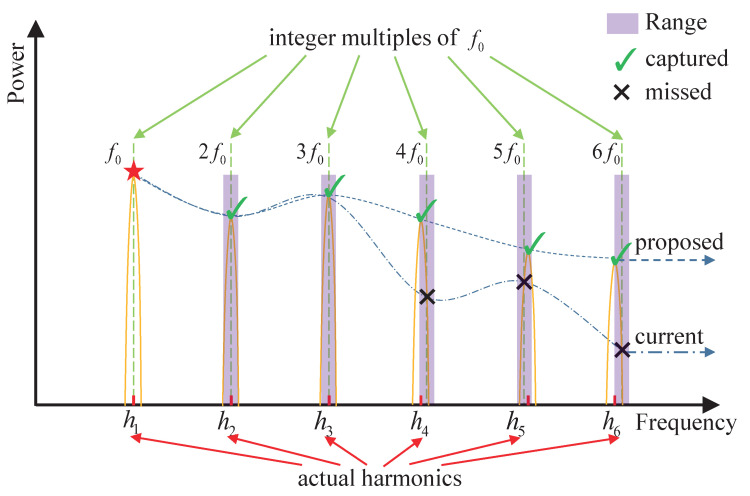
Modeling harmonic structure with loose constraint.

In the proposed method, the strict integer multiple constraint of harmonic frequencies in (5) is modified by adding an adjustable parameter Δf to capture harmonics more accurately. The harmonic frequency $f_{l}$ is selected in a small range that is controlled by Δf:

(6) $$P(f_{l})=\max\left(P\left(lf_{0}-\Delta f:lf_{0}+\Delta f\right)\right),l=2,\ldots L$$

where $f_{l}$ is the frequency of the harmonic of order number *l*, and *L* is the maximum order of harmonic to be considered. The expression max(P(x:y)) represents the maximum power FFT spectrum within the frequency range from *x* to *y*. Therefore, when summing the harmonic power, the proposed method does not select the frequency bin at the integer multiple position as in the SRH, but searches in a frequency range from $lf_{0}-\Delta f$ to $lf_{0}+\Delta f$. The frequency deviations of actual harmonics are small, and the deviations are enlarged to demonstrate the capture effect of the proposed modeling method on harmonics, as shown in Figure 4. The frequency range is determined by the order number *l* and parameter Δf. The order number *l* controls the primary harmonic position, and parameter Δf determines the allowable deviation degree of harmonic frequency relative to the integer multiple position. A large value of Δf or *L* decreases the accuracy because too loose of a constraint could capture spectral peaks that do not belong to the harmonic. Experiments show that setting Δf as two frequency bins is a good balance between the accuracy and the loose constraint. Two frequency bins correspond to 2 Hz since the frequency resolution in this paper is 1 Hz. In the case of lower frequency resolution, two frequency bins are still reasonable. Because leakage is the primary factor affecting the capture of harmonics, the two frequency bins cover the effects of different types of windowing operations. Besides, since the first few significant harmonics contain most of the energy of the speech signal, *L* is set to 5 in the proposed method.

In Figure 4, symbols, such as $h_{1}$, $h_{2}$, $h_{3}$, etc., on the horizontal axis represent the peak positions of the actual harmonic frequencies, and the corresponding peaks on the vertical axis represent the harmonic spectrum. Symbols $f_{0}$, 2$f_{0}$, 3$f_{0}$, etc. in the upper position denote the frequencies with a strict integer multiple relationship. The symbols “*√*” and “×” are the intersections of the vertical dashed lines and the harmonic peaks. The fourth to sixth harmonics in the spectrum slightly deviate from the position of integer multiples of the pitch. The purple shading shows the allowable frequency deviations of the proposed method. The mismatch between the symbols “×” and the harmonic peaks indicates that the current modeling method on the harmonic structure cannot accurately capture all the harmonics in this situation. The coincidence of the symbol “*√*” and the harmonic peak represents the effectiveness of the proposed method.

### 3.3. Segment Extension

**Figure 5:**
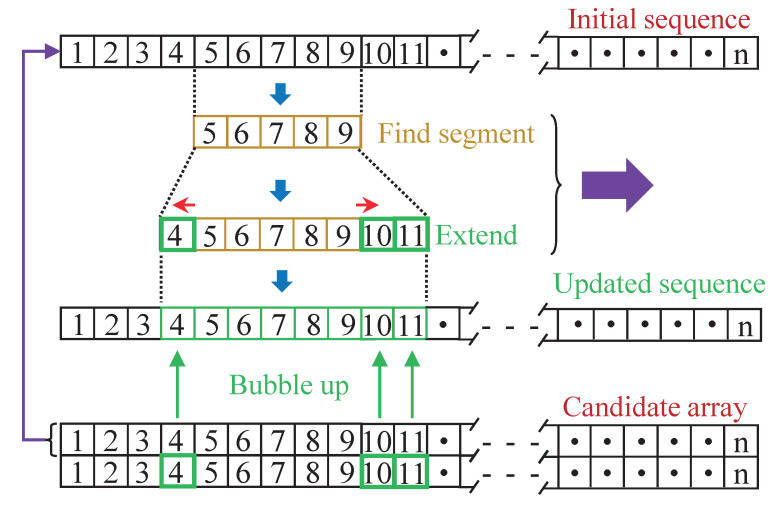
Block diagram of the main pitch segment extension.

The first row of the candidate array is selected as the initial sequence, as is shown in the top of Figure 5, and the numbers 1, 2, 3, …, n in Figure 5 indicate the frame index. The initial sequence is used to find the main segment of the pitch because the frequencies within the first row of the candidate array are most likely to be the pitch from the perspective of the SRH value. Based on the smooth prior of the pitch trajectory, the parameter $f_{\mathrm{shift}}$ is utilized to control the maximal frequency shift between frames. If the frequency change of two adjacent frames Δf is less than the size controlled by the parameter $f_{\mathrm{shift}}$, the two adjacent frames are considered as continuous frames, as follows:

(7) $$\left\{\begin{array}{c} \Delta f(i)=\mathrm{abs}(f(i+1)-f(i))\\ \Delta f\left(i\right)\leq f_{\mathrm{shift}}\times f\left(i\right) \end{array}\right.$$

where *i* denotes the index of the initial sequence. Parameter $f_{\mathrm{shift}}$ is critical because it should balance the continuity and the changing trend of the pitch trajectory. Parameter $f_{\mathrm{shift}}$ is set to 0.11 in the proposed pitch estimation method. This value is initially guided by statistical analysis of the TIMIT pitch dataset [32], and then fine-tuned by experiments. The value of 0.11 is small for the pitch range. For the 100 Hz pitch, the frequency difference is only 100 × 0.11 = 11 Hz. Therefore, increasing the frequency resolution is necessary to distinguish the tiny frequency difference, and the frequency resolution in the proposed method is set to 1 Hz.

The minimal length of the main pitch segment in this paper is set to 140 ms, corresponding to 5 frames for the selected parameter setting. If other hop size hopSize is specified, the minimal number of consecutive frame ncF needs to be adjusted by:

(8) $$ncF=\mathrm{ceil}(\frac{140-dF}{hopSize})+1$$

where ceil(x) represents the function of rounding the elements of *x* to the nearest integers towards infinity, and dF denotes the data frame length. The main pitch segment consisting of frames 5 to 9 illustrates how the extension is performed on the main pitch segment in the middle portion of Figure 5. The main pitch segment is extended forward and backward by searching frequencies from the candidate pitch array, and the frequency that satisfies (7) is added at the corresponding end of the main pitch segment in turn. Frames 4 to 12 constitute an example of the extension segment, which is then used to replace the corresponding segment of the initial sequence and form the updated sequence. After that, forward and repeat the “find segment” and “extend” processes in the initial sequence until its end.

**Figure 6:**
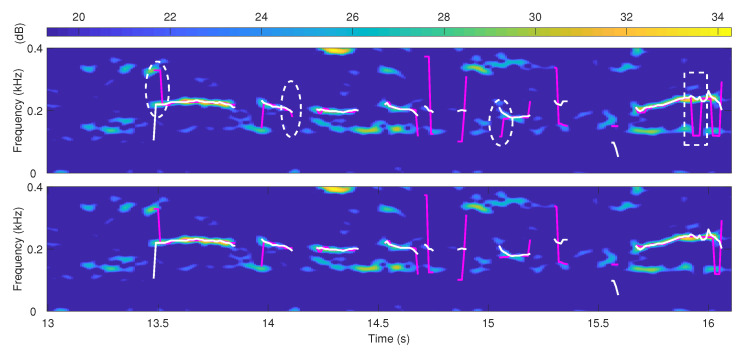
Pitch estimation of the proposed method without the segment extension approach (the upper) and with the segment extension approach (the lower). The spectrogram and the ground truth correspond to a segment of speech signal named “f1nw0000.pes” in the Keele database. The white lines are ground truths, and the red lines are the estimated results. The speech is mixed with babble noise of −5 dB SNR.

Figure 6 shows how segment extension improves the performance. The improvement mainly includes two aspects highlighted by the circle dashed boxes and the square dashed boxes. The circle dashed box parts reflect the improvement for two pitch segment ends, where the error jumps are reduced by extending the correct pitch segment. The square dashed box part represents the improvement for the errors in the middle of the pitch segment, where the main pitch segment extension scheme enables most correct estimates to suppress short-term error estimates.

### 3.4. Voiced/Unvoiced Decision

A small modification to the voiced/unvoiced decision of the original SRH method is made and integrated into the proposed method. This subsection will first introduce the scheme of the voiced/unvoiced decision in the original SRH method, and then realize the modification.

The original SRH method realizes the voiced/unvoiced decision through two SRH thresholds. Based on the fact that the SRH value of the voiced segment is higher than that of the unvoiced segment, the original SRH method judges the SRH values corresponding to the pitch sequence, and generates voiced/unvoiced decisions for threshold values higher or not higher than the threshold. This threshold is set to 0.07 in the code. Besides, a threshold adjustment is realized by judging the standard deviation of the SRH values. When the standard deviation exceeds 0.05, the threshold 0.07 is increased to 0.085. This concept of double threshold is inherited also in the proposed method. The threshold adjustment is beneficial to improve the adaptability to different SNR conditions. Because a larger standard deviation means a higher SRH dispersion degree, and a higher SRH dispersion degree generally means a higher SNR.

**Figure 7:**
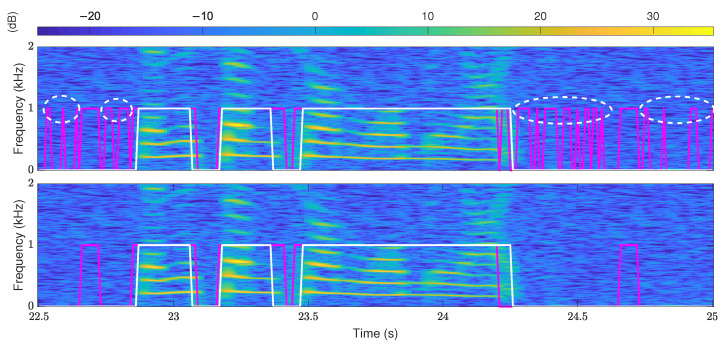
Voiced/unvoiced decision results of the original SRH method (the upper) and the proposed method (the lower). The spectrogram and the ground truth correspond to a segment of speech signal named “f1nw0000.pes” in the Keele database. The white lines are ground truths, and the red lines are the estimated results. The speech is mixed with white noise of 10 dB SNR.

The above voiced/unvoiced decision lacks the consideration of continuity, which leads to temporary errors. As shown by the upper one in Figure 7, many temporary voiced decisions that differ from the ground truth are short-term errors. The modification handles the temporary errors by adding a judgment on the voiced/unvoiced decision results. The time of consecutive voiced decisions is checked, and the voiced decisions that are less than a time threshold are modified as unvoiced. The time threshold is set to 140 ms, which is the same as the threshold of the main pitch segment. The results of the modified voiced/unvoiced decisions are shown by the lower one in Figure 7. It can be seen that the modified voiced/unvoiced decision effectively reduces multiple short-term errors circled by the white dotted lines.

## 4. Evaluation

The proposed pitch estimation method is compared with ten pitch estimation methods under noise and reverberation conditions. This section first introduces the methods, datasets, and evaluation metrics used for performance comparison, and then analyzes the results.

### 4.1. Comparison Methods and Dataset

#### 4.1.1. Comparison Methods

The comparison methods include the SRH [14], HS-based methods such as SHR [17], widely referenced methods such as YIN [22], PEFAC [18], RAPT [23], YAAPT [25], SWIPE [24], neural network-based CREPE [30], and state-of-the-art fast NLS (here it refers to the version with Bayesian feature) [12,19]. The source codes of the comparison methods used in the experiments are listed in Table 1, wherein the source code of the proposed method is also listed to make it free for public use. All the codes are in the Matlab version. Besides, the source codes are generally selected from the links provided by the original authors of the methods, the Matlab websites, and the links widely cited by related research.

#### 4.1.2. Dataset

The datasets used in the evaluation include three portions: the pitch dataset, the noise dataset, and the reverberation dataset. The pitch dataset adopts the widely used Keele database [33]. It contains six minutes of ten spoken sentences from five male and five female speakers, all of whom read a phonetically balanced text from Aesop’s Fable “The Northwind and The Sun”. The audio is a 20 kHz sampling rate and 16-bit resolution, and the ground truth is recorded from the electroglottography. The speech and audio signal lengths are 0.04 h and 0.06 h, respectively. The time increment of the ground truth is 10 ms, and the frame length used is 25.6 ms.

The noise dataset is obtained by adding white noise and four color noises in the NOISE-X92 noise dataset to the clean pitch dataset according to different SNRs. The audio parameters of the NOISE-X92 noise dataset are 19.98 kHz sampling rate and 16-bit resolution [34]. The reverberation dataset is obtained by convolving the clean pitch dataset with 24 room impulse responses (RIRs) generated using the image source method [35]. The parameter setting for generating RIRs by the image source method is given in Table 2, where RT60 represents the time it takes for a sound to decay by 60 dB, and quantifies the severity of room reverberation. A higher RT60 means a more severe reverberation.

### 4.2. Accuracy Evaluation

#### 4.2.1. Evaluation Metrics

The metrics used in the evaluation include the gross pitch error (GPE), mean absolute error (MAE), and voiced decision error (VDE), which assess the accuracy of the pitch estimation methods from different aspects.

GPE is the most important and most widely used accuracy evaluation metric for pitch estimation, and it is defined as the percentage of the estimated pitches that deviate from the ground truth by a threshold *p*. Though the common setting of the threshold *p* is 0.2, the thresholds *p* = 0.1 and *p* = 0.05 also exist [15]. The GPE results corresponding to the three thresholds of *p* are all given in the evaluation. The GPE calculation method in this evaluation experiment is as follows:

(9) $$\mathrm{GPE}=\frac{\sum_{n}〚\Delta f\left(n\right)\geq p\wedge v\left(n\right){〛}_{I}}{\sum_{n}〚v\left(n\right){〛}_{I}}$$

where $〚\cdot {〛}_{I}$ represents the logic operations and outputs 0 or 1 accordingly. The symbol “∧” denotes the “and” logical operation, and is used to filter out the data required for the metric calculation. Assume the estimated pitch sequence and the ground truth pitch sequence are $f_{\mathrm{estimation}}\left(n\right)$ and $f_{\mathrm{truth}}\left(n\right)$, respectively. The range of the sequence index *n* is 1≤n≤N. ΔF represents the degree of the estimated pitch that deviate from the ground truth, and is calculated by:

(10) $$\Delta f\left(n\right)=\left|\frac{f_{\mathrm{estimation}}\left(n\right)-f_{\mathrm{truth}}\left(n\right)}{f_{\mathrm{truth}}\left(n\right)}\right|$$

The calculation method of GPE is inconsistent in v(n) in different literature. Specifically, the difference is to consider the frame that the ground truth is voiced and the estimated pitch is unvoiced, or not. The two conditions correspond to (11) and (12), respectively.

(11) $$v\left(n\right)=[f_{\mathrm{truth}}\left(n\right)\neq 0]$$

(12) $$v\left(n\right)=\left[f_{\mathrm{estimation}}\left(n\right)\neq 0\wedge f_{\mathrm{truth}}\left(n\right)\neq 0\right]$$

This paper adopts (11) when the ground truth is voiced and the estimated pitch is unvoiced. Because a lower GPE can be achieved if the confidence threshold for judging voiced segment is set higher in (12), and this affects the fairness of GPE comparison.

The unvoiced frames of the ground truth are given as 0s in the Keele dataset [33], and these frames are excluded when calculating the metrics of the pitch estimation methods. Besides, the pitches of the voiced frames that are wrongly labeled as unvoiced frames by different pitch estimation methods are set to 0 Hz.

MAE reflects the accuracy of pitch estimation through the degree of frequency deviation, and MAE is calculated as follows:

(13) $$\mathrm{MAE}=\mathrm{mean}\left(|f_{\mathrm{estimation}}\left(n\right)-f_{\mathrm{truth}}\left(n\right)|\wedge v\left(n\right)\right)$$

VDE is the error percentage of the voiced/unvoiced decisions for all audio frames. The VDE criterion is employed for voiced/unvoiced segment decision, and VDE is calculated by:

(14) $$\mathrm{VDE}=\frac{\mathrm{sum}\left(v_{\mathrm{estimation}}\left(n\right)\neq v_{\mathrm{truth}}\left(n\right)\right)}{N}$$

where $v_{\mathrm{estimation}}\left(n\right)$ and $v_{\mathrm{truth}}\left(n\right)$ denote $n^{\mathrm{th}}$ estimated value and ground truth, respectively.

#### 4.2.2. Experimental Results

Noise experiments include white and colored noise conditions under SNR from −15 dB to 20 dB with a step size of 5 dB. The reverberation experiments are carried out under different RT60s. Note that there is a flag for configuring the whitening preprocessing in the input of the comparison method fast NLS. Since the fast NLS method does not provide an automatic switching logic of the flag, the flag is configured as “1” throughout the experiment. The detailed experimental results are introduced separately below.

**Figure 8:**
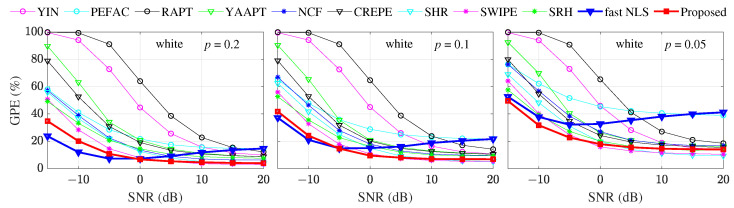
GPE comparison under white noise, different threshold *p*, and different SNRs.

(1) The GPE results under the white noise conditions are shown in Figure 8, where the bold red lines with “□” symbols represent the proposed method. The green lines with “*” symbols and the bold blue lines with “▿” symbols represent the SRH and the fast NLS methods, respectively.

Figure 8 shows similar patterns of the GPE curves for most methods as below.

- The GPE tends to be a small value at high SNRs, and the GPE rises rapidly when the SNR is negative. This is consistent with the fact that the pitch is easier to extract at high SNRs.
- Except for the fast NLS, the GPE of different methods are less different under high SNR conditions, but are significantly different under low SNRs.
- The curve of fast NLS does not always go lower when the SNR increases. This result is different from other methods, but is consistent with the experimental results in the fast NLS paper [12]. The reason may be that the whitening operation of the fast NLS influences the harmonic structure of the speech signals.
- For a specified method under a specified SNR condition, the smaller the threshold *p*, the higher the GPE. Such as the proposed method at 0 dB SNR, the GPEs are about 8%, 11%, and 20% for threshold *p* = 0.2, *p* = 0.1, and *p* = 0.05, respectively. This also holds for the proposed method at 0 dB SNR, where the GPEs are about 8%, 11%, and 20% for threshold *p* = 0.2, *p* = 0.1, and *p* = 0.05, respectively.

Except for the above overall conclusions, specific conclusions about the pitch estimation methods are as follows.

**Figure 9:**
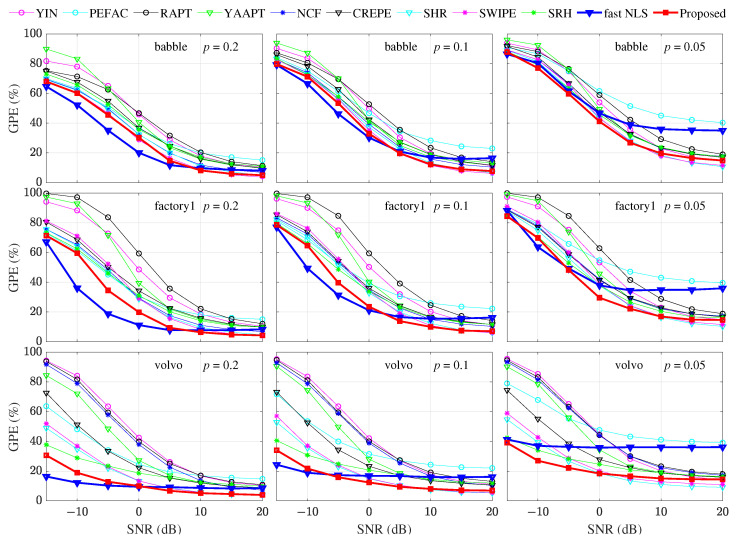
GPE comparison under colored noise, different SNRs, and different thresholds of *p*.

**Figure 10:**
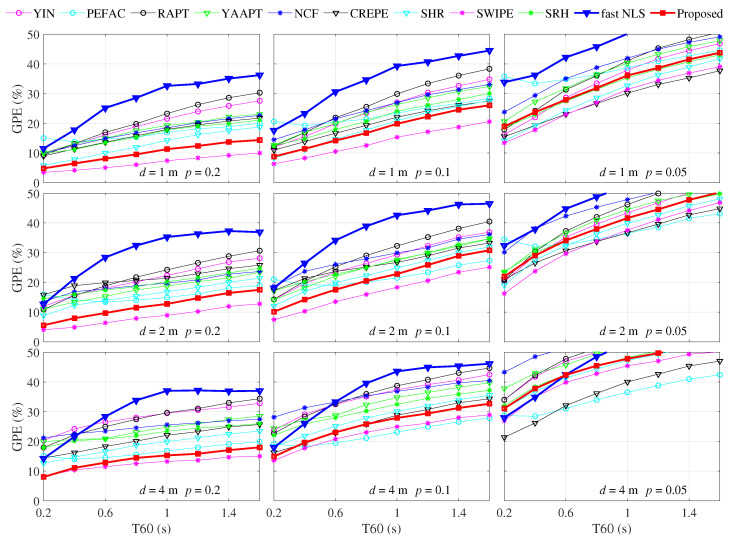
GPE comparison under different reverberation conditions and different thresholds of *p*.

- The proposed method maintains close to the lowest GPE curve (state-of-the-art fast NLS) under negative SNRs and *p* = 0.2 and *p* = 0.1. However, the gap between the fast NLS and the proposed method becomes smaller as the accuracy requirement of pitch estimation increases (smaller *p*), and the proposed method outperforms the fast NLS when *p* = 0.05.
- The proposed method still maintains close to the lowest GPE curve (SWIPE) under positive SNRs, and this GPE advantage of the proposed method is kept for all *p* thresholds. The largest difference between the GPE of the proposed method and the lowest GPE occurs at *p* = 0.05, SNR = 20 dB, where the difference is only about 5%.
- The GPE curve of fast NLS first decreases with the increase of the SNR, and then shows an upward trend with the increase of the SNR. The inflection point of the downward and upward trend tends to lower SNRs as the threshold *p* decreases. When the SNR is higher than 5 dB, the error rate of fast NLS is even higher than the proposed method.

(2) The GPE results under four colored noise conditions are shown as twelve sets of results in Figure 9. Every three sets of results of each row correspond to the same type of colored noise, and every four sets of results of each column correspond to the same threshold *p*.

- For all methods, under the same colored noise and SNR conditions, the effect of the threshold *p* is still such that a smaller threshold *p* corresponds to a higher GPE.
- Compared with fast NLS, for different colored noises, under *p* = 0.2 and low SNR conditions, the GPE of the proposed method is close to the fast NLS with the lowest GPE, and the proposed method gradually shows an advantage over the fast NLS as the SNR increases.
- Compared with fast NLS, for a specified colored noise, the performance gap between the proposed method and fast NLS gradually decreases as threshold *p* decreases, and under the conditions of *p* = 0.05, the proposed method exhibits an obvious advantage.
- Compared with the methods except the fast NLS, under high SNR conditions, the difference is very small between the proposed method and the optimal method. Moreover, under low SNR conditions, the GPE of the proposed method is generally lower than all methods.

(3) The GPE results under different speaker to microphone distances *d*, different RT60s, and different threshold *p* are shown in Figure 10. In the nine GPE results, every three sets of results of each row corresponds to the same speaker to microphone distance, and every three sets of results of each column correspond to the same threshold *p*. To show the overall trend of GPE, the vertical axis scales are set to range from 0% to 50%.

The nine sets of results demonstrate the accuracy change of all the pitch estimation methods from high accuracy to near failure caused by the reverberation and stricter accuracy requirement. The upper leftmost result corresponds to the slightest reverberation and the loosest accuracy requirement, and the overall trend of GPE is lower. On the contrary, the bottom right corner result is under the most severe reverberation and the most strict accuracy requirement, and the overall trend of GPE is higher.

- For the same speaker-to-microphone distance *d*, the smaller the threshold *p*, the higher the GPE.
- For the same threshold *p*, the longer the speaker-to-microphone distance *d*, the higher the GPE. This is probably because the proportion of direct sound decreases as the distance *d* increases.
- Under the conditions of *p* = 0.2 and *p* = 0.1, the SWIPE method maintains apparent advantages, and the performance of the proposed method is closest to the SWIPE compared with all other methods. Besides, the advantages gap between the proposed method and the SWIPE method gradually decreases as the speaker-to-microphone distance *d* increases.
- Under the condition of *p* = 0.05, the CREPE performs best, and the proposed method is in the middle level of all the methods.

(4) The MAE comparison of the proposed method and the comparison method under noise and reverberation conditions is shown in Table 3 and Table 4, where the MAE results are the average values under the four noise types and three distance *d* conditions, respectively. The results in blue color denote the optimal one under the condition of the corresponding column. In general, the trend of MAE of different methods is similar to that of GPE: as SNR increases, MAE tends to a small value; as RT60 increases, MAE increases. Besides, the MAE of the proposed method is optimal in most reverberation and noise conditions. Under noisy conditions, the MAE of the proposed method remains the lowest in the SNR range from −15 dB to 20 dB, which shows that the average estimation accuracy of the proposed method is the highest. Under reverberant conditions, the MAE of the proposed method remains the lowest in the range of RT60 = 0.2 s to RT60 = 1 s. From RT60 = 1.2 s to RT60 = 1.6 s, the MAE difference between the proposed method and the lowest SWIPE method is negligible.

In conclusion, the optimal GPE results do not correspond to one fixed pitch estimation method when the noise, reverberation, and threshold *p* conditions vary. The overall GPE performance of the proposed method is the best. Because the proposed method exhibits the best GPE performance under a sizable proportion of conditions, it approaches the best GPE performance under many conditions.

(5) The voiced/unvoiced decision comparison between the proposed method and the original SRH method under noise and reverberation conditions is shown in Table 5 and Table 6. Overall, the proposed method’s voiced/unvoiced decision accuracy is superior to the original SRH method in almost all experimental conditions. The improvement under most conditions is within 5%, and the improvement under some conditions is close to 10%. Besides, the improvement degrees under noise conditions are generally higher than that under reverberation conditions, which indicates that the proposed method has a certain inhibitory effect on noises. Very few results show no improvement, whose voiced/unvoiced decision errors are close to 50%, and the comparison has become meaningless.

### 4.3. Time Cost Evaluation

#### 4.3.1. Evaluation Metrics

The time cost evaluation is conducted by comparing a metric named run time ratio of different methods on a same computer. The run time ratio Γ is defined as the ratio of the time cost $T_{\mathrm{method}}$ of one method on the computer to the actual time $T_{\mathrm{actual}}$ of the audio signal, as shown in (15).

(15) $$\Gamma =\frac{\sum T_{\mathrm{method}}}{\sum T_{\mathrm{actual}}}$$

a lower Γ value means a lower time cost ratio and a better performance. Besides, a Γ value great than 1 represents that the time cost of the method is longer than the actual time of the audio signal on the computer, and a Γ value less than 1 means that the time cost of the method is shorter than the actual time of the audio signal on the experimental computer.

The time cost evaluation is affected by the number of samples, and the accuracy of results can be improved more effectively through multiple statistics and the average of the time cost $T_{\mathrm{method}}$. Besides, the time cost $T_{\mathrm{method}}$ of the proposed method is affected by the sampling rate of the dataset. The time cost of the proposed method in this evaluation is based on the sampling rate of 20 kHz.

The primary setup of the computer is as follows:

- Intel(R) Core(TM) i5 6500 CPU @ 3.2 GHz;
- x64-bit-based Windows 7 operating system;
- DDR4 RAM with 16 GB capacity;
- SSD hard-disk of 256 GB capacity;
- AMD Radeon R5 430 graphics board with 2 GB of video memory.

#### 4.3.2. Experimental Results

The time costs of different methods vary greatly, and the corresponding Γ values are shown in Table 7 in descending order. The following conclusions can be obtained from Table 7, and all the descriptions are based on the experimental computer.

- The proposed method’s time cost is about one-half the actual audio signal time.
- The time cost of the proposed method is about one-eighth (3.83/0.49) of the state-of-the-art fast NLS.
- The time cost of the NCF method is the lowest among all the methods, which is consistent with the simple calculation and structure of the method itself.
- The time costs of the fast NLS and CREPE are rather high on the computer used for the experiments because the time costs are 3.81 times and 6.93 times the actual audio signal time, respectively.

## 5. Discussion

The long-term median significantly increases the output latency of the proposed method, but there is much room for improvement. A possible improvement is replacing the long-term median with a short-term median of a speech segment updated over time. As a result, the long-term median is generated shortly after the onset of the speech signal, and the output latency of the subsequent pitch estimates is significantly reduced.

The voiced/unvoiced decision accuracy of the proposed method does show superior performance to the original SRH method. However, it is likely that the proposed segment extension approach still has the potential to further improve voiced and unvoiced decisions. The segment expansion is currently used to “narrow” voiced/unvoiced decisions. Similarly, the segment expansion is likely to be used to expand voiced/unvoiced decisions, and this is the approach that we are investigating.

## 6. Conclusions

The modeling methods of harmonic structure used by current pitch estimation methods do not always show consistency with the actual harmonic distribution of the signal. The inconsistency decreases the accuracy of pitch estimation methods. Starting with the summation of residual harmonics (SRH) method, this paper makes two improvements to it. First, the method of modeling harmonic structure is revised to have a loose constraint. Therefore, the shifted harmonics can be captured. Second, a high-efficiency smooth scheme named main pitch segment extension is integrated into the SRH to reduce the short-term errors of the pitch segment. Further, the idea of pitch segment is also applied to the SRH’s voiced/unvoiced decision to remove short-term errors. Accuracy experiments with ten pitch estimation methods verify that the proposed method has better overall accuracy and robustness. Specifically, the proposed method outperforms the state-of-the-art fast NLS method under a considerable proportion of experimental conditions. Time cost experiments show that the time cost of the proposed method reduces to around one-eighth of the fast NLS method on the computer used for the experiments. 

## Figures and Tables

**Table 1:** The source code links of the pitch estimation methods.

Method	Source Code Link (Accessed on 6 June 2022)
SHR	https://www.mathworks.com/matlabcentral/fileexchange/1230
SRH	https://www.mathworks.com/help/audio/ref/pitch.html
PEFAC	http://www.ee.ic.ac.uk/hp/staff/dmb/voicebox/voicebox.html
YIN	http://audition.ens.fr/adc/sw/yin.zip
RAPT	http://www.ee.ic.ac.uk/hp/staff/dmb/voicebox/voicebox.html
YAAPT	http://www.ws.binghamton.edu/zahorian/yaapt.htm
SWIPE	https://github.com/kylebgorman/swipe
CREPE	https://ssd.mathworks.com/supportfiles/audio/crepe.zip
NCF	https://www.mathworks.com/help/audio/ref/pitch.html
fast NLS	https://github.com/LimingShi/Bayesian-Pitch-Tracking-Using-Harmonic-model
Proposed	https://github.com/deshengwang001/SRH_Variant

**Table 2:** Parameter setting of the image source method.

Parameter Name	Number	Value
room size	1	[5 × 6 × 3] (m)
speaker position	1	[1,1,2] (m)
microphone position	3	[1,2,2] (m), [1,3,2] (m), [1,5,2] (m)
RT60	8	0.2 s–1.6 s, step size is 0.2 s

**Table 3:** MAE comparison in Hz under noise conditions.

SNR (dB)	−15	−10	−5	0	5	10	15	20
YIN	127.89	117.77	93.65	63.48	40.27	26.44	19.74	16.87
PEFAC	81.27	69.89	55.30	41.62	33.17	29.07	27.66	26.90
RAPT	138.43	129.71	104.79	71.26	44.54	28.69	20.67	16.96
YAAPT	137.76	120.04	85.80	53.65	31.53	21.65	16.89	14.88
NCF	117.76	103.95	80.44	53.00	32.18	20.20	14.49	12.04
CREPE	103.50	83.09	58.98	41.10	29.34	22.45	18.59	16.24
SHR	67.67	56.62	41.35	26.91	17.01	10.92	7.65	6.08
SWIPE	76.28	65.83	49.98	31.90	19.25	11.38	7.77	6.17
SRH	81.33	68.97	53.90	39.92	29.20	22.08	18.35	16.66
fast NLS	56.55	32.91	21.52	15.71	13.14	12.70	12.60	12.82
Proposed	37.46	22.65	13.70	9.98	8.47	7.97	7.36	6.99

**Table 4:** MAE comparison in Hz under reverberation conditions.

RT60 (s)	0.2	0.4	0.6	0.8	1.0	1.2	1.4	1.6
YIN	24.64	29.18	31.97	33.87	35.49	36.83	38.08	39.25
PEFAC	27.70	24.40	24.30	24.65	26.01	26.68	27.82	28.76
RAPT	18.22	22.56	25.57	28.07	30.51	32.61	34.54	36.08
YAAPT	20.51	22.44	23.13	25.26	26.55	27.96	29.15	30.61
NCF	24.79	27.33	28.22	28.75	29.49	30.09	31.21	31.58
CREPE	23.12	25.55	27.31	29.06	31.04	32.63	34.26	35.44
SHR	12.43	14.87	16.92	18.73	20.43	21.90	23.31	24.38
SWIPE	9.01	10.38	11.60	12.53	13.61	14.26	15.15	15.75
SRH	24.72	27.82	29.19	30.48	33.25	34.66	35.41	36.80
fast NLS	13.87	20.78	28.52	32.63	35.27	35.99	36.08	36.30
Proposed	7.96	9.37	11.05	12.32	13.87	14.82	15.72	16.40

**Table 5:** VDE comparison in percentage under noise conditions.

SNR (dB)		−15	−10	−5	0	5	10	15	20
white	SRH	38.15	31.5	26.6	24.64	22.43	16.71	12.56	12.69
	proposed	32.84	23.5	16.95	14.8	13.45	11.01	9.03	9.18
babble	SRH	47.64	47.12	45.91	44.37	42.1	39.46	37.23	27.94
	proposed	48.05	46.92	45.47	43.05	39.62	36.34	33.59	21.77
factory1	SRH	47.56	44.45	41.2	35.26	31.96	31.21	23.45	17.71
	proposed	48.54	42.47	37.98	29.51	25.08	23.25	17.27	12.14
volvo	SRH	31.95	26.99	24.31	18.3	15	12.35	12.16	12.44
	proposed	26.16	20.8	17.93	13.96	11.13	9.41	9.13	9.28

**Table 6:** VDE comparison in percentage under reverberation conditions.

RT60 (s)		0.2	0.4	0.6	0.8	1	1.2	1.4	1.6
d = 1 (m)	SRH	29.62	40.03	41.15	41.78	42.32	42.44	42.83	43.28
	proposed	24.16	37.35	38.5	39.42	39.97	40.2	40.97	41.51
d = 2 (m)	SRH	28.68	40.4	42.1	42.76	43.28	43.61	43.82	44.29
	proposed	22.38	37.42	39.52	40.51	41.17	41.6	42.12	42.84
d = 4 (m)	SRH	38.15	43.19	43.37	43.78	43.77	44.26	44.28	44.53
	proposed	34.44	41.55	41.54	41.71	42.09	42.43	42.97	43.04

**Table 7:** The ratio of the cumulative time cost of a pitch estimation method to the cumulative actual time of the audio signal.

Method	Γ	Method	Γ
CREPE	6.93	YIN	0.18
**fast NLS**	3.83	YAAPT	0.12
**Proposed**	0.49	SHR	0.1
RAPT	0.48	PEFAC	0.08
SRH	0.13	SWIPE	0.07
		NCF	0.01

## Data Availability

Not applicable.

## Abbreviations

The following abbreviations are used in this manuscript:

CREPE	Name of pitch estimation method: Convolutional Representation for Pitch Estimation
DSR	Distributed speech recognition
ETSI	European elecommunications Standards Institute
FFT	Fast Fourier transform
GPE	Gross pitch error
GMM	Gaussian mixture model
HS	Harmonic summation
Keele	Pitch estimation dataset
LPC	Linear predictive coding
MAE	Mean absolute error
NLS	Non-linear least squares
NCF	Name of pitch estimation method: Normalized Correlation Function
NCCF	Normalized cross-correlation function
PEFAC	Name of pitch estimation method: Pitch Estimation Filter with Amplitude Compression
RIR	Room impulse response
RT60	Reverberation time 60
RAPT	Name of pitch estimation method: Robust Algorithm for Pitch Tracking
SNR	Signal-to-noise ratio
SRH	Name of pitch estimation method: Summation of Residual Harmonics
SHR	Name of pitch estimation method: Subharmonic-to-Harmonic Ratio
SWIPE	Name of pitch estimation method: Sawtooth Waveform Inspired Pitch Estimator
TIMIT	Pitch estimation dataset
YIN	Name of pitch estimation method
YAAPT	Name of pitch estimation method: Yet Another Algorithm for Pitch Tracking
$A_{l}$, $a_{l}$	Linear weight of the $l^{\mathrm{th}}$ harmonic
*d*	Speaker to microphone distance
e(n)	$n^{\mathrm{th}}$ sampling of noise
$f_{0}$	Pitch
$f_{l}$	Frequency of $l^{\mathrm{th}}$ harmonic
$f_{s}$	sampling rate
$f_{\mathrm{median}}$	Long-term pitch median
$f_{\mathrm{estimation}}$	Pitch estimation
$f_{\mathrm{truth}}$	Pitch ground truth
$f_{\mathrm{shift}}$	Maximum frequency difference between pitch of adjacent frames
$f_{0,\min}$	Minimum value of the frequency search range of pitch estimation
$f_{0,\max}$	Maximum value of the frequency search range of pitch estimation
$h_{1},h_{2},h_{3},...$	Actual harmonic frequencies
*L*	Maximum order of harmonic
*l*	Order of harmonic
*P*	Residual power spectrum
*p*	Threshold of maximum frequency deviation percent
Range	Frequency search range of pitch estimation
$R_{n}$	Signal to noise ratio at the input port
$T_{\mathrm{actual}}$	Time of actual audio length
$T_{\mathrm{method}}$	Time cost of pitch estimation method
*u*	Mean of the tied GMM
$X_{k}$	Noise figure of actual gain control
$x_{n}$	$n^{\mathrm{th}}$ sampling of signal
Δf	Frequency difference
$\omega_{0}$	Normalized angular frequency in radians
